# Provincial policies affecting resident quality of life in Canadian residential long-term care

**DOI:** 10.1186/s12877-023-04074-y

**Published:** 2023-06-09

**Authors:** Pamela Irwin, Deanne Taylor, Janice M. Keefe

**Affiliations:** 1 Nova Scotia Centre on Aging, Mount Saint Vincent University, Halifax, NS Canada; 2 Interior Health Authority, Kelowna, BC Canada; 3 Rural Coordination Centre of British Columbia, Vancouver, BC Canada; 4 Department of Family Studies and Gerontology, Mount Saint Vincent University, Halifax, NS Canada

**Keywords:** Long-term care, Policies, Resident, Quality of life, Risk mitigation

## Abstract

**Background:**

The precautions and restrictions imposed by the recent Covid-19 pandemic drew attention to the criticality of quality of care in long-term care facilities internationally, and in Canada. They also underscored the importance of residents’ quality of life. In deference to the risk mitigation measures in Canadian long-term care settings during Covid-19, some person-centred, quality of life policies were paused, unused, or under-utilised. This study aimed to interrogate these existing but latent policies, to capture their potentiality in terms of positively influencing the quality of life of residents in long-term care in Canada.

**Methods:**

The study analysed policies related to quality of life of long-term care residents in four Canadian provinces (British Columbia, Alberta, Ontario, and Nova Scotia). Three policy orientations were framed utilising a comparative approach: situational (environmental conditions), structural (organisational content), and temporal (developmental trajectories). 84 long term care policies were reviewed, relating to different policy jurisdictions, policy types, and quality of life domains.

**Results:**

Overall, the intersection of jurisdiction, policy types, and quality of life domains confirms that some policies, particularly safety, security and order, may be prioritised in different types of policy documents, and over other quality of life domains. Alternatively, the presence of a resident focused quality of life in many policies affirms the cultural shift towards greater person-centredness. These findings are both explicit and implicit, and mediated through the expression of individual policy excerpts.

**Conclusion:**

The analysis provides substantive evidence of three key policy levers: situations–providing specific examples of resident focused quality of life policy overshadowing in each jurisdiction; structures–identifying which types of policy and quality of life expressions are more vulnerable to dominance by others; and trajectories–confirming the cultural shift towards more person-centredness in Canadian long-term care related policies over time. It also demonstrates and contextualises examples of policy slippage, differential policy weights, and cultural shifts across existing policies. When applied within a resident focused, quality of life lens, these policies can be leveraged to improve extant resource utilisation. Consequently, the study provides a timely, positive, forward-facing roadmap upon which to enhance and build policies that capitalise and enable person-centredness in the provision of long-term care in Canada.

**Supplementary Information:**

The online version contains supplementary material available at 10.1186/s12877-023-04074-y.

## Background

For many older Canadians, a long-term care (LTC) facility becomes their end-of-life home. Among the oldest age group, 28 per cent of Canadians aged 85 years and over, lived in a LTC residence in 2021 [1].

Historically, LTC policies in Canada have prioritised biomedically-oriented quality of care [2], but over time, a culture change towards a more person-centred model of care has evolved [3]. Additional research [4–7], among others, has indicated that a *home-like* approach to care creates more opportunities for residents’ overall quality of life (QoL) and wellbeing [8, 9]. Accordingly, many LTC oriented policies now reflect a broad spectrum of person-centered QoL indicators, for instance, early research describing 11 domains, such as meaningful activity, relationships, dignity, autonomy, and privacy [10, 11].

However, during the Covid-19 pandemic, many person-centred policies were superseded by the risk prevention and amelioration priorities of care providers in LTC settings in Canada [12, 13]. Since then, collected research [14–18] suggests that some of these policies continue to be paused, unused, and/or under-used in the Canadian LTC environment.

Consequently, this study aimed to conduct an in-depth analysis of these latent pre-Covid policies to capture their potentiality to positively influence the QoL of LTC residents in Canada.

## Literature review

A recent review of international LTC determined that the scope of services, funding sources, types of ownership, and regulatory requirements and enforcement practices, varied greatly across residential settings and countries [19]. Although Canada was not included in this study, the Canadian LTC landscape is also characterised by diversity, encompassing private, non-profit, and public funding models, government and private ownership, and mixed regulation and enforcement regimes [20]. These differences may be attributed (in part) to the legislative and jurisdictional boundaries operating in Canada. The Canadian federal government does not insure LTC as part of the Canada Health Act, although it does provide funding to the provinces to help offset this cost. Consequently, individual provinces and territories have jurisdictional responsibility for LTC oversight and administration, resulting in variation within and across provinces and regions [20, 21]. Within Canada [22] and internationally [19], this complexity “highlights the absence of a cross-national understanding of quality care and a minimum standard of provision. This is in contrast to…a shared understanding of the value of person-centred approaches and settings that support person-centered care.” [19 p.6] The balance between resident quality of care and quality of life in LTC in Canada is underscored in a new standards publication [109], that advocates for greater resident self-determination embedded in a resident-centred care environment.

The concept of person-centred care has developed over time, ranging from patient-focused care [23]; patient-centred care [24–27]; and relational care [28, 29]; to theoretical advances in person-centred care [2, 30, 31]; and more recently, person-directed care planning in nursing homes [32]. Even though this continuum of care represents a broad canvas of approaches, models, and elements, all subscribe to a central tenet: “High quality person-centered care is focal for residents.” [19 p.6].

Conversely, attempts to disentangle the relative contributors of person-centred care and resident characteristics in LTC have not been as decisive. Two systematic reviews [2, 33] were inconclusive; but a later study [34] linking resident and facility factors to QoL in LTC was more definitive, concluding that “helping residents maintain functional abilities and providing an engaging social environment may be particularly important in improving quality of life.” [34 p.643].

This finding is supported in seminal work on resident QoL in LTC [10, 11]. In addition to the domains of functional competence and meaningful activity [34], sense of safety, security, and order; physical comfort; enjoyment; relationships; dignity; privacy; individuality; autonomy/choice; and spiritual wellbeing are also recognized [10, 11]. Related research [35–38] expands these determinants of QoL in residential care, while complementary literature addresses QoL from the residents’ point of view [39]. For example, “Satisfying residents’ preferences and providing a greater range of choices may result in greater quality of life.” [40 p.184] This approach is reflected in various policy documents mandating resident satisfaction surveys in LTC facilities in Canada [41], and the rise of preference-based, person-centred models of care [42–48]. These initiatives rest on a premise that that LTC residents value a sense of control and empowerment over their life choices [10, 11, 44, 45, 47, 49–51].

Despite this cultural shift in LTC policies and practices, residential facilities are fraught with “circumstances that direct attention towards physical care and organisational needs at the expense of residents’ overall wellbeing.” [50 p.4] A recent study of residential care homes determined that space and time pressures negatively impacted the care staff provided to LTC residents, with implications for hygiene and infection control standards, plus more interpersonal “virtues” such as dignity and respect, [52 p.1] where “the ‘invisible’, emotional, and immeasurable aspects of care, largely became the first to be relinquished.” [53 p.66].

The competing priorities between quality of care versus QoL at different levels of influence in Canada have been documented [22], in particular. These perspectives are extended through additional research at an organisational level probing staff perceptions of the tension between resident centred care and quality [54], and comparing institutional regulation and standardisation versus the subjective practices of care workers [55]; and deconstructing the interface between physical and emotional safety [56], and individual freedom or dependence [57], in the context of residents’ perceptions.

In 2015, the term *surplus of safety* was coined to represent the prevailing approach to risk in LTC settings [58]. Subsequent papers [16, 59, 60] supported this premise by highlighting the dominance of safety, security, and orderliness in Canadian LTC settings. Furthermore, updated guidance [61], reviews [62], and research-informed reports [63–65] into the impact of the Covid-19 pandemic on residents of LTC facilities, reached similar conclusions.

While respecting the overarching emphasis on risk mitigation, this prioritisation essentially relegates other person-centred QoL policies to a secondary status–in effect, creating a cadre of existing, but under-utilised policies. Consequently, this study aimed to interrogate these latent (pre-Covid-19, person-centred, QoL focused) policies to capture their potentiality in terms of positively influencing the QoL of residents in LTC settings in Canada. It is based on a sub-set of the Seniors–Adding Life to Years (SALTY) project–a large pan-Canada research initiative consisting of four linked streams. Stream four investigated federal and provincial level policies that enable or hinder resident QoL in LTC facilities in British Columbia, Alberta, Ontario, and Nova Scotia, through four lenses– staff [59], family [16], volunteer [60], and in this paper, a resident-specific focus.

## Method

There are many approaches to policy analysis [66–68]. However, these methodologies are not primarily purposed to locate latent (existing, but un-utilised, and under-utilised) QoL domains in resident related LTC policies, whereas a novel method of policy analysis [69] was developed and implemented [59, 60] to embrace this (and related) objectives. As a result, it provided a rigorous platform upon which to base the analytical processes.

Grounded in two complementary theories, objective hermeneutics [70] and content analysis [71], and predicated on the 11 QoL domains [10, 11], the method [69] is also underpinned with expert and user groups [72] to provide monitoring of the process and validation of the findings. In brief, it comprises four iterative and inter-related stages involving an in-depth interpretive examination of the meaning of language used in LTC related policy documents: stage one, policy collection; stage two, policy categorising; stage three, policy ranking; and stage four, policy selection.

A foundational team of three researchers located relevant policy documents, devised preliminary coding criteria, and piloted potential analytical sequences. These processes were continually member-checked with a primary investigator, with revisions implemented according to the team consensus. For example, early decisions advocated including only policy texts related to older people *resident* in LTC facilities and exempted policies about accessing LTC (see Supplementary Figs. 1 and [69]). Further coding involved alignment of the selected policies with one or more of the 11 QoL domains–safety, security, and order; physical comfort; food/enjoyment; meaningful activity; functional competence; relationships; dignity; privacy; individuality; autonomy/choice; spiritual well-being [10, 11].

As noted above, this research was part of a four-year project, extending from 2016 to 2020, with policies collected until the end of 2017. However, to minimise data contamination, it was decided to stop adding and/or recoding any new policies during the policy categorising, ranking, and selection stages (between 2018 and 2019). Despite these constraints, the initial research identified 350 potential documents that pertained to policies affecting residents of LTC facilities.

**Fig. 1:**
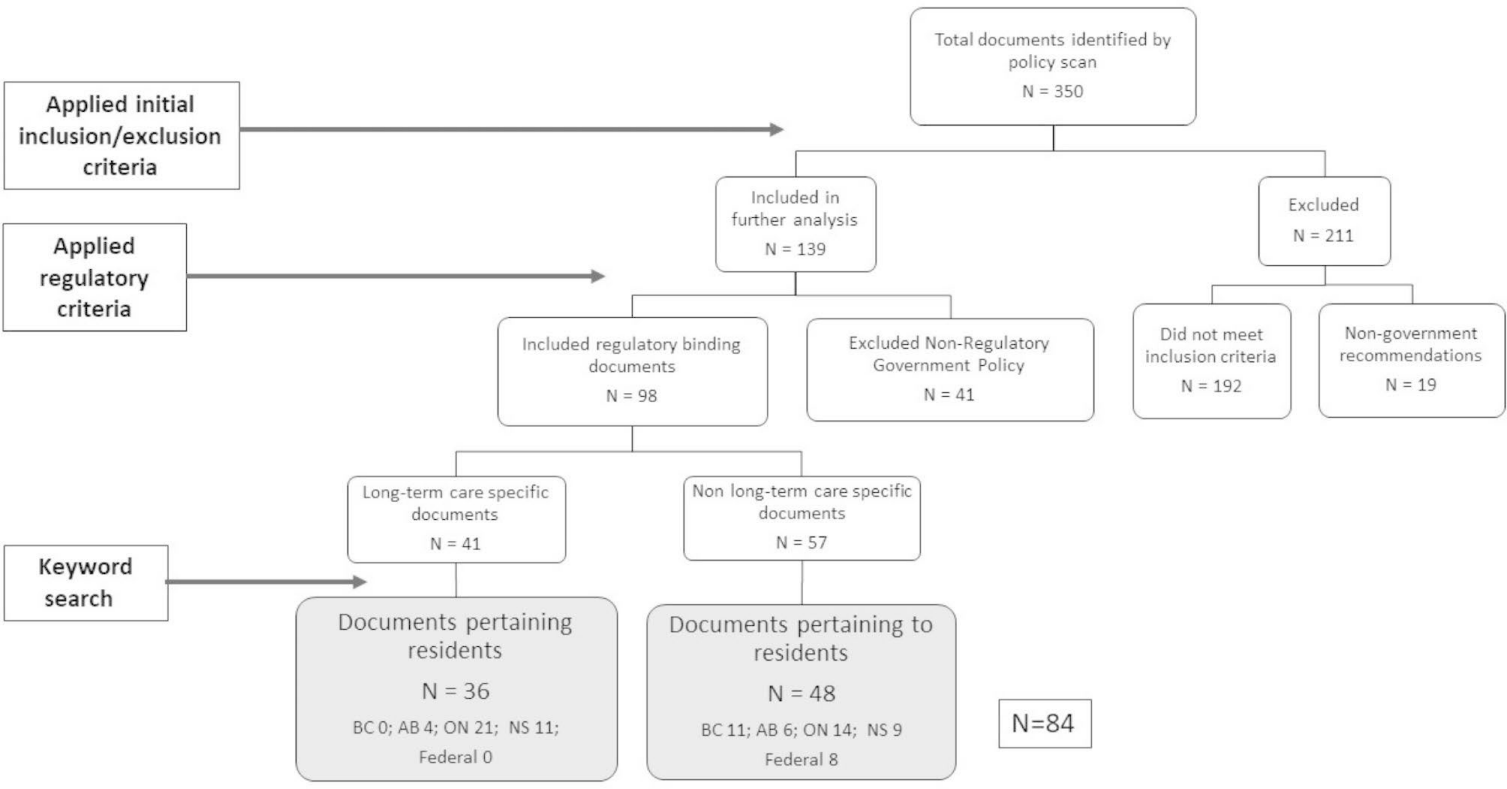
Policy Inclusion and Classification Process for Resident-related Policies in LTC

Since the method required a complex, in-depth textual analysis of each policy, additional screens were needed to reduce the volume of relevant documents. Various filters aided this process, for example, the application of inclusion and exclusion criteria, developed in conjunction with an overarching advisory group (Supplementary Fig. 1). Policies were included if they pertained to residents of LTC facilities aged 65 years and over, and were classified as influential provincial or federal documents. (N = 192, Fig. 1). However, policies created or released after July 2017, and identified as non-binding (strategies, best practices from non-governmental organisations, professional associations, non-profit agencies, advocacy groups (N = 19, Fig. 1) were also excluded. The binding versus non-binding criterion differentiated ‘*would like to do’* (a union or advocacy group is not obligated to implement an in-house strategy); from ‘*should do’* (a strategy for change proposed by a provincial Ministry or Health Authority that is not yet required N = 41), to *‘must do’* (legislation, and/or regulation that mandates accountability for meeting specific conditions such as infection prevention and control, staff ratios, and fire codes). Subsequent guidance from the advisory group advocated inclusion of the *must comply* (highest degree of obligatory power) LTC related polices only (Fig. 1), and resulted in a library of 139 policies. (See [69] for complete details.)

Next regulatory criteria were applied. Policies were divided into those regulated by government (N = 98) including LTC specific policies (N = 41) and non-LTC specific policies (N = 57), or strategies authored by government but not regulated (N = 41). These non-regulatory policies were omitted (see Fig. 1).

Further restrictions were then applied to develop a resident-specific policy library. A series of key word search terms (person, client, patient, resident) was used to determine a resident specific baseline. This process yielded 84 polices. 36 policies were specific to LTC and related to residents’ wellbeing, with another 48 polices pertaining to residents, but were not specific to the LTC environment, such as building and fire codes, and food handing policies for congregate settings.

A final step categorised policies according to the authority proscribed by the policy (federal and provincial governments). Of these, eight policies were created by the federal government of Canada, and the remaining 76 were from British Columbia, Alberta, Ontario, and Nova Scotia. (Note: for clarity, details about each policy are recorded in the accompanying supplementary tables rather than in the reference list. All of these policies are in the public domain and accessible via respective provincial government websites. However, when individual policies are specifically cited in the text, they are fully referenced. It is also important to note that all policies were collected and collated during 2017, and since then, some have been amended and updated).

## Analytical approach

**Fig. 2:**
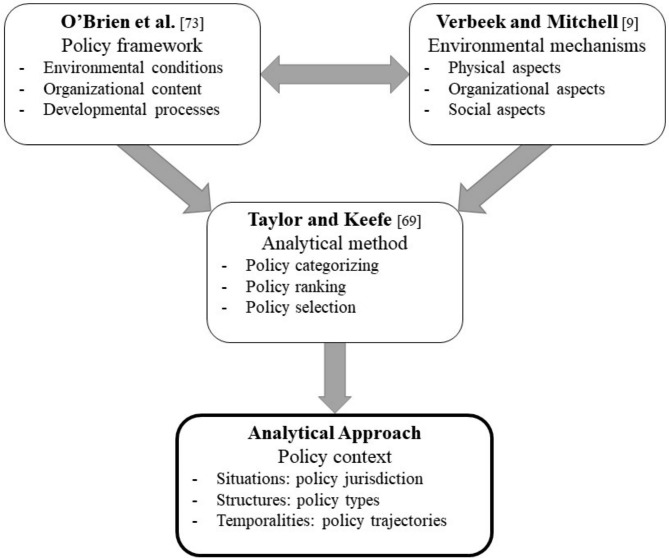
Conceptual and Methodological Origins of the Analytical Approach Applied in this Research

Although previous research [59, 60] utilised initial analyses, additional conceptual development was required to sharpen the analytical focus for the resident lens. This new, resident-centred locus resulted in a reformulated analytical approach, represented in Fig. 2, and expanded below:

One adaptation involved orienting the analytical process with specific contextual prisms. A comprehensive review of methodological approaches to policy synthesis and analysis revealed many approaches [66–68], among others. To enhance methodological rigour and consistency within and across analytical processes, the election of a compatible approach rested on the four stages identified previously, namely documentary collection, categorisation, ranking, and selection. The final inter-related, tiered approach was informed by two analytical frameworks–one centered on health-related policy analysis [73, 74], and the other, contextualised to LTC settings [9]. The health policy triangle framework [73, 74] offers a wide utility in terms of health contexts, systems, and issues [73], and rests on three apices, namely: environmental conditions (context–systemic factors: social, economic, political, cultural, and other environmental conditions); organisational content (structures: operational policies, legislation, regulations, guidelines); and developmental process (how policies are initiated, developed or formulated, negotiated, communicated); in effect, situations, structures and temporalities respectively. These paralleled three key environmental mechanisms (physical aspects, organisational aspects, and social aspects) that underpin the LTC spectrum [9]–chosen to reflect the broad canvas of the current LTC environment.

An integrative, textual (content) synthesis [75, 76] was used for the synthesis. The synthesis of the frameworks realised: situations–environmental (physical) conditions; structures–organizational content; and trajectories–developmental (social/cultural) processes. Here, it is important to note that the four iterative stages described in the method (policy collection, categorising, ranking, and selection) were layered within both the synthesis [75 pp.736–737, 76 p.500] and analytical processes.

The next step involved articulating the outcome of the synthesis (situations, structures, and trajectories) with the analytical method [69]. Specifically designed to systematically account for a multitude of polices across diverse jurisdictions, systems, and sequences, this method enabled an integrated approach to analysing the policies, leading to: policy categorising–situational focus; policy ranking–structural focus; and policy selection–temporal focus, for this analysis. These focal refinements guided the following in-depth jurisdictional (situational), policy types (structural), and trajectory (temporal) analyses.

This approach also involved methodically condensing and configuring the volume of resident related policies into manageable assemblages. Thus, for policy categorisation, policies were cross-referenced according to jurisdiction, regulatory level, and relevant QoL domains (Supplementary Table 1). LTC specific or non-specific LTC policies were also listed (see Supplementary Tables 2 and 3 respectively), and also by types and jurisdiction to facilitate policy (structural) ranking; and the evolution and development of policies over time (policy selection) were traced with reference to Supplementary Table 1. A recursive, iterative process was used to analyze *all* policy excerpts for each focus, with indicative examples drawn from the most applicable policies.

Similarly, to address QoL, policies listed in Supplementary Tables 2 and 3 were cross referenced with the QoL domains identified in Fig. 2. These policies were then examined at the intersection of type and QoL domains. And while each domain was analysed in turn, three domains were chosen as exemplars of the findings– safety, security and order to align with its dominance in the literature, and relationships and individuality to capture different aspects of person-centred care.

At this juncture, it is timely to reiterate that the intent of analysis was to examine the paused, unused, and/or underused QoL policies that are overshadowed by more dominant policies such as safety, security, and order, rather than negating the importance of risk mitigation interventions. The next section describes the findings using the approach (Fig. 2 and outlined above) which includes policy situations, policy types, and policy temporality (or how the policies changes over time).

## Findings

### Situational (jurisdictional) focus

The situational finding describes the influence of policies based on which jurisdiction the policy is applied. An initial scan of Supplementary Table 1 suggests that the policies tend to coalesce into three groups: policies with numerous QoL domains, those encompassing few domains, and some falling in an approximate mid-range. This broad categorisation is not intended to represent the significance or impact of these policies–the Federal policies are a case in point. Although policies created by the Federal government represent a relatively limited number of QoL domains, their influence on the care landscape across Canada is extensive. And as noted above, although all policies and policy excerpts were analyzed, the majority of the citations relate to policies with the most QoL domains.

**Fig. 3:**
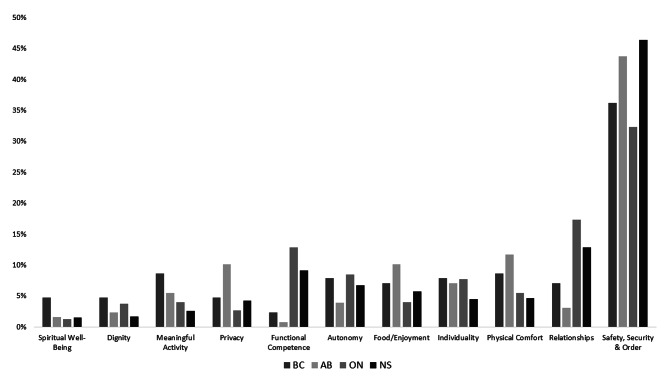
Proportion of LTC Policy Excerpts by QoL Domains located within Resident-related LTC Policies by Province

Examination of the policies and relevant QoL domains revealed that some policy excerpts explicitly prioritized safety, security, and order over other QoL domains (Fig. 3).

For instance, in Ontario: “Every resident has the right to keep and display personal possessions, pictures and furnishings in his or her room subject to safety requirements and the rights of other residents” [41], thereby potentially constraining a resident’s individuality, autonomy and choice. Similarly, in Nova Scotia, the 2017 Building Code regulations [77] require “Where there are more than 20 suites, a closed-circuit visual monitoring system shall be provided capable of connection to individual suites”, with implications for residents’ privacy.

Contrary to expectations from the literature, the preceding analysis indicates that QoL curtailment is also present in other policy domains, not only safety, security, and order. This finding represents a diffuse assemblage of QoL prioritization across all jurisdictions. Here, selected examples include:

- food/enjoyment: “The operator of a long-term care accommodation shall ensure that the menu provided for residents as far as is reasonably practicable….” [78]; and “Residents are encouraged to eat in the dining room. Alternate arrangements based on residents’ needs may be made, provided there is adequate supervision” [79];

- relationships: “There is no restriction on visitors except when: requested by the residents or their authorized designates; a visitor is deemed by the licensee to pose a security risk or to negatively impact other residents or the operations of the home…” [79];

- autonomy/choice: “Every licensee of a long-term care home shall ensure that each resident of the home…is dressed appropriately, suitable to the time of day and in keeping with his or her preferences, in his or her own clean clothing and in appropriate clean footwear.” [80]; and “Residents are encouraged to personalize their bedrooms with their personal possessions in a manner that is safe and practical” [79], with concomitant impacts on residents’ expressions of individuality in each instance.

Some policies are apparent contradictions. On one hand, the Alberta Design Guidelines for Continuing Care Facilities [78] indicate that:

The resident bedroom is the resident’s “own” [private] space; an area where they can do as they please…Accordingly, the bedroom is familiar to the resident, which may be facilitated by having some of their own personal furniture (e.g., dresser, desk, easy chair or small entertainment unit) in their room.

and on the other:

Bedrooms are organized and sized to facilitate quality resident care which may include the provision of direct care by one or more caregivers/staff, simultaneously…Caregivers/staff require unobstructed access to the bed to deliver care to a resident, while in bed.

leading to a potential conflict between residents’ individuality and staff access if furniture is very large.

In addition, policies that reduce residents’ access, and hence autonomy, in managing their own financial affairs are evident in Alberta “An operator shall return funds held in trust to the resident or the resident’s representative on receiving a request in writing to do so.” [81] and Nova Scotia [82]:

The long-term care facility must establish policies for the management of the resident trust accounts, including posted hours when resident trust account funds are available, amounts that may be withdrawn in cash, and notice that is required for larger withdrawals.

Related research suggests that the less instrumental *soft* QoL domains, including dignity, individuality, autonomy and choice, and spiritual well-being, may be more likely to be displaced by instrumental *hard* domains such as safety, security, order, and functional competence [16, 59, 60]. Again, this tendency was not fully realised, whereby a *soft* domain (autonomy and choice) potentially overrode another–individuality (reported above).

These unanticipated findings invited a closer examination of the jurisdiction, policy, and QoL interface. The following analysis reveals the role of language and terminology in tempering the expression of QoL policies. As above, various permutations were identified.

Some policies in Alberta and Nova Scotia stand out for their use of very direct terms, such as “elopement” in both the Alberta Design Guidelines for Continuing Care Facilities [78]: “The (bedroom) window cannot open more than 152 mm (6 inches) to avoid elopement”, and the Nova Scotia Long Term Care Facility Requirements Space and Design [83]: “Each resident house must have the ability to be secured to prevent resident elopement.”

Other policies display a normative approach, with terms like “appropriate” in Alberta and Ontario, and “reasonable”, “reasonably expected” in British Columbia: “A licensee must ensure that each bedroom, bathroom and common room is lit sufficiently to permit a person to carry out effectively the types of activities that would be reasonably expected in the ordinary use of the room.” [84].

Presumptive or *leading* statements are employed in some Ontarian policies: “meals in congregate settings unless resident needs indicate otherwise” and “Mood and behaviour patterns, including wandering, any identified responsive behaviours, any potential behavioural triggers and variations in resident functioning at different times of the day.” [80] Conditional qualifiers (“may”, “subject to”) are also embedded in some British Columbian and Ontarian policies:

Every licensee of a long-term care home shall ensure that each resident of the home receives an offer of an annual dental assessment and other preventive dental services, subject to payment being authorized by the resident or the resident’s substitute decision-maker, if payment is required [80].

A comparison of the terminology used in operational-type policies in Ontario and Nova Scotia reveals differences as well. Ontario adopts a prescriptive orientation, for example, specifying the thickness of mattresses on residents’ beds [80], whereas Nova Scotia presumes a more nudge phraseology mediated through suggestions prefaced with “consider”:

Consider wheelchair and walker maneuverability, as well as resident gait when selecting floor finishes, to ensure that residents can move about the facility safely (e.g., carpets can present difficulties for residents with gait/walking problems, and can create undue resistance for residents confined to wheelchairs) [83].

Finally, the overarching style of language foregrounded in the policies is suggestive of the purpose and structures of the policy – which also points at how varied jurisdictions apply policies. For instance, some policies are prefaced by aspirational premises: “Affirm our commitment to preserving and promoting quality accommodation that provides a safe, comfortable, home-like environment and supports a high quality of life for all residents of long-term care homes” [41] and “The physical environment must support a holistic approach to resident centered care – addressing physical, social, mental and spiritual well-being” [83], whereas others are clearly more operational and prescriptive in nature [80]. As yet, it is not established if there is a connection between the purpose of the policy and predominance of specific QoL domains. Consequently, the next phase of this analysis (policy ranking) deconstructs the structural attributes (type) of policies against QoL domains to determine the presence of any that are under-utilised.

### Structural (types of policy) focus

Policy types include regulations, standards, manuals, and guides, as listed in Supplementary Tables 2 and 3. An analysis of these elements portrays a diffuse picture of the types of policies at play.

There are no LTC specific policies generated by the Federal or British Columbia governments. Of the remaining jurisdictions, policies in Alberta are broadly focused on standards related to quality of care; most policies in Ontario comprise financial and/or funding supplements to the core Long Term Care Homes Act 2007 [41]; and a policy mix of special care [85] and special needs [86] acts, and space and design requirements [83] represents Nova Scotian policies. Furthermore, with the exception of Alberta, all Federal, Ontarian, Nova Scotian, and the majority of policies in British Columbia, are non LTC specific regulations. Policies in Alberta encompass regulations, standards, and guidelines.

Regulations in Nova Scotia provide a representative example of the non LTC specific and exclusive LTC parallelism: the Long Term Care Facility Requirements Space and Design 2007 [83] requires that “the physical environment must provide opportunities for meaningful relationships, interactions and companionship with residents, family, staff and the community”; whereas the Homes for Special Care Act Regulations 2012 [87] states that “every home for special care shall have suitable space, both indoors and outdoors apart from bedrooms, for the relaxation of the residents and reception of visitors.”

As detailed previously, non LTC and exclusive LTC policies encompass different types of policy document, and thus, different purposes. Again, these differences are reflected in the authority of the language.

In general, regulations focus on compliance via directives such as “must”: “The records for all residents of a home for special care must be kept in a safe and secure location and must be accessible at all times to the supervisory staff of the home and to inspectors.” [87] Some of these regulations are prefaced with mandated principles and statements of resident rights: “Every resident has the right to be properly sheltered, fed, clothed, groomed and cared for in a manner consistent with his or her needs.” [41].

Standards are also voiced as requirements, incorporating “shall ensure” terms in Alberta: “An operator shall ensure that each resident of a long-term care accommodation has the opportunity to personalize the resident’s room.” [88] Alternatively, standards in British Columbia are characterised by more malleable language: “recognises and accommodates residents’ preferred bedtimes, awakening times and other sleep/rest routines.” [89].

Manuals too, may be pitched as mandates as in Nova Scotia’s Special Needs Policy– Long Term Care 2008 [86]: “Items or services purchased as a special need will be competitively priced, cost-effective and appropriate for the resident.” This terminology is also evident in Ontario’s Long Term Care Home Design Manual 2015 [90]: “Each washroom must be designed to promote resident privacy, dignity and independence. In addition, the washroom space must also allow caregivers to provide effective and safe care delivery.” However, these “must” statements contradict the preceding aspirational principle that promotes flexibility:

The Design Manual continues to promote innovative design in long-term care homes in Ontario, by giving service providers flexibility to create environments that make it possible to respond positively and appropriately to the diverse physical, psychological, social and cultural needs of all long-term care home residents [90].

A similar mismatch occurs for guidelines in Alberta. The flexible Design Guidelines for Continuing Care Facilities 2014 [78] state that where “The bedroom is also the resident’s “own” space…” and “The bedroom is familiar to the resident, which may be facilitated by having some of their own personal furniture (e.g., dresser, desk, easy chair or small entertainment unit) in their room”, while the later Accommodation Standards and Licensing Information Guide 2015 [81] indicates that: “An operator shall ensure that each resident of a long-term care accommodation has the opportunity to personalize the resident’s room.”

These findings highlight some jurisdictional differences related to the types (purpose) of policy, as evidenced through representative terminology. For instance, conditional terms such as “may”, found in the British Columbia Model Standard for Continuing Care and Extended Care Services 1999 [89], are the antithesis of the formal authoritativeness of the Canada Health Act 1985 [91]. And excepting the dissonance noted within some policies above, this analysis suggests that policies differ in their *steadfastness*–depending on their purpose (type of policy). Not unexpectedly, regulations and standards are more rigid than manuals and guidelines, and concomitantly, different types of policy signal different levels of mutability. While it is feasible that policies in manuals and guidelines are susceptible to curtailment by the less elastic regulations and standards, it is not yet established if QoL domains also exert an effect.

The relationships between policy types and QoL domains are examined as follows, but firstly, to recapitulate briefly: although the analysis addressed each QoL domain in turn, three domains were chosen as exemplars of the findings–safety, security and order, relationships, and individuality. Here too, it is helpful to recognize that not all policy types are represented in each QoL domain.

In general, regulations related to the safety, security and order QoL domain are grounded in ‘rights-type’ principles, and precise “will not”, “must”, and “shall ensure” compliance-oriented terminology. The Alberta Long Term Care Standards and Checklist 2010 [88] typify safety standards: “the operator shall ensure that heating, cooling and ventilation systems are operated at a level that maintains a temperature that supports the safety of all residents and the comfort of the majority of the residents.” Manuals for LTC also embrace “must” type terms, in addition to “provide” and “offer” as in the British Columbia Home and Community Care Policy Manual Chap. 6 Residential Care Services 2016 [92]:

Health authorities must ensure service providers plan and manage the change process for clients where a service provider is planning a large scale staff replacement…ensure that maintenance of the quality and safety of the client’s care is the priority throughout the process; provide the client with information about the upcoming change; offer clients and families an opportunity to meet with service provider staff to identify the key concerns in the changeover in staff.

Similarly, for the relationship domain, regulations encompass “entitled to” and “shall have” wording. Standards specific to LTC focus on strict “shall ensure” terminology, but non LTC specific policies, such as the British Columbia Model Standard for Continuing Care and Extended Care Services 1999 [89], may be more person-centered: “During residents’ admission and orientation, the interdisciplinary team welcomes residents, caregivers, familiarizes them with their surroundings, and introduces them to residents and staff representatives.” Likewise, supportive terms such as “enabling” are evident in LTC specific manuals: “The beauty salon/barber shop enables residents to participate in an enhanced level of grooming that is a familiar activity of daily living.” [90] and “Residents should be encouraged to manage their own assets or personal funds” [82].

By contrast, the authoritative tone of regulations for the individuality domain is somewhat muted in Nova Scotia: “A residential style window in the wall between the resident bedroom and the corridor promotes resident choice to see into common areas when in bed or in a chair.” [83] and in British Columbia: “Until the contrary is demonstrated, every adult is presumed to be capable of making decisions about personal care, health care and legal matters and about the routine management of the adult’s financial affairs.” [93] Standards too, enable individuality in non-LTC specific policies: “recognises and accommodates residents’ preferred bedtimes, awakening times and other sleep/rest routines.” [89] Individuality is expressed as facilitation in LTC manuals, and guidelines in non-LTC specific settings: “An operator shall ensure that each resident of a long-term care accommodation has the opportunity to personalize the resident’s room.” [81].

These examples propose that for different QoL domains, policies embracing more permissive terminology (“offers”, “opportunity”) are at risk for supplanting by policies denoting more instrumental terminology such as safety, security, and order.

Overall, the intersection of policy types and QoL domains supports the previous finding that some policies, particularly safety, security and order, may be prioritised over other QoL domains. Two trends strengthen this perspective:

- compared with regulations and standards (instrumental policy types), manuals and guidelines tilt towards person-centredness;
- compared to the more instrumental QoL domains such as safety, security, and order,

individuality, dignity, and spiritual wellbeing are more relational and person-centred.

Building on the preceding analyses, it then follows that policies in manuals and guidelines that focus on relational QoL domains may be susceptible to restriction by more instrumental policies–in this context, regulations and standards related to safety security and order. Here, the Ontario Long Term Care Home Design Manual 2015 [90] provides a cogent illustration: “Residents have the opportunity to see and smell food, snacks can be prepared and residents can make food choices at the point of meal service.” It is not difficult to foresee the many circumstances under which this “opportunity” could be constrained.

However, with respect to the culture shift to person-centred LTC, it is not known if the trends observed above are an artifact of this change. The influence of temporal changes on policies is explored next, with Supplementary Table 1 serving as a reference point.

### Temporal (policy trajectory) focus

A chronological examination of the policies in British Columbia revealed that the language referring to residents of care facilities changed from “resident” [89], to “person in care” [84], and finally, “client” [92]. This trajectory towards instrumentalisation is antithetical to the wider movement of person-centredness in LTC settings, and calls for further analyses.

Accordingly, a group of policies was selected for each province in turn. With the exception of the 2008 Long Term Care Policy in Nova Scotia [86], all policies were regulations.

The Revised Statutes of British Columbia RSBC 1996 [94] included the Mental Health Act c288 and Representation Agreement Act c 405. A Pharmacy Operations and Drug Scheduling Act SBC 2003 c 77 was added in 2003, and a Seniors Advocate Act SBC 2013 c 15 in 2013 [95]. The seniors advocate has a “Duty to advise on seniors’ issues…in an independent manner, the minister, public officials and persons who deliver seniors’ services on systemic challenges faced by seniors, on policies and practices respecting those challenges” [95]. This policy is a tangible recognition of older person rights.

In Alberta, the core Nursing Homes Act 1985 [96] included General Regulations 232, and an enabling statute RSA 2000 c N-7 in 2000.

As part of life enrichment services, an operator of a nursing home shall, in accordance with a resident’s wishes, grant access to a person representing a religion to meet with the resident in the nursing home and to hold a religious service in an appropriate place in the nursing home, and where practicable, an operator shall encourage and assist residents to leave his nursing home to visit, shop and attend religious services and community activities [96].

Numerous supplements to the Ontario Long Term Care Homes Financial Policy 2010 [97] are listed in Supplementary Table 1. Broadly, these address operational, fiscal, and service provision changes to the core regulation. Although most of these additions are procedural, they also include some elements of person-centredness. For instance, the Long Term Care Homes Financial Policy Level of Care per Diem 2013 [98] focuses on improving the quality of care by relaxing some regulatory constraints, such as funding staff training, dietary consultations, and incontinence supplies. These modifications also positively impact resident QoL. Similarly, funding for nurse practitioners in LTC facilities enhances both resident quality of care and QoL:

“Participates in creating an organizational environment that supports the safety, quality of resident care and life, collaborative practice” and “Engages with the resident in regular dialogue about their care plan; Utilizes communication and counselling skills: Engages residents in dialogue to determine what is important to them for health and quality of life.” [99]

Additions to the Special Needs Policy–Long Term Care 2008 in Nova Scotia [86] include Over Cost Fund in 2008; Resident Trust Accounts in 2009; HELP Specialized Equipment Program in 2014 [100]; and Resident Charge in 2016. While these policies are predominantly prescriptive, detailing specific conditions for funding and service provision, they are individually tailored to resident needs: “Items or services purchased as a special need will be competitively priced, cost-effective and appropriate for the resident” [86]; and specialised equipment in the HELP program is prescribed by a physiotherapist or occupational therapist [100].

Although quality of care is prioritized over QoL across these policies, the additional enhancements mark a tacit recognition of various aspects of person-centredness. As represented here, the cultural shift towards person-centred care is scattered and mosaic-like, rather than a holistic developmental trajectory, with two notable exceptions:

The Alberta Resident and Family Councils Act 2017 [101] focuses specifically on improving, maintaining, and enhancing all aspects of residents’ quality of life:

The purposes of a resident and family council are…to provide a forum for the residents and their families to discuss ways of maintaining and enhancing the residents’ quality of life in the residential facility… to provide opportunities for the residents and their families to develop and participate in projects for the residents’ benefit…to provide a network of support and encouragement for the residents and their families.

Provincial design guidelines for care facilities are also oriented towards person-centredness. This is reflected in the synergistic requirements for residents’ bedrooms in Alberta: “Each bedroom should have “cueing” features, (e.g., a familiar objects/pictures), outside the bedroom, within the corridor, to assist residents in finding their way and identifying their bedroom” [78]; Ontario: “The resident bedroom is the centre of a resident’s personal space. Its design must meet the resident’s need for comfort and safety, promote the resident’s independence and provide for resident privacy” [90]; and Nova Scotia: “Bedroom configuration and planning should permit personalization through multiple bed locations” in each room.” [83].

Taken together, this analysis demonstrates that polices evolve over time through the use of language (the resident descriptors in British Columbia); adding supplements to core policies; and creating special policies to address a specific purpose. Although many of these initiatives are not specifically oriented towards person-centred care, various person-centred QoL domains are supported throughout the policies. The building design guidelines in particular, offer a way towards an optimised resident QoL.

## Summary

The overarching aim of analysis was to uncover dormant, unused, and/or under-used resident related QoL polices in LTC facilities in British Columbia, Alberta, Ontario, and Nova Scotia. Accordingly, the systematic and sequential analysis of the 84 resident-focused, LTC related policies indicated that QoL is expressed differently depending on situational, structural, and temporal influences. When policies were categorised according to jurisdiction, regulatory type, and relevant QoL domains, some policy excerpts explicitly prioritised safety, security, and order over other QoL domains. Other policies were similarly at risk of displacement, but this effect was implicit and tempered by the differential use of language/terminology. For policy ranking, policies were structured according to policy types and jurisdictions. This ranking uncovered the connection between the purpose of the policy and predominance of specific QoL domains. Two key findings were revealed: policies in manuals and guidelines are susceptible to curtailment by the less elastic regulations and standards; and for different QoL domains, policies embracing more permissive terminology are less resistant to dominance compared with policies denoting more instrumental terminology such as safety, security, and order. Finally, the temporal analysis identified that policies evolved through language, supplementation, and generally in line with the cultural shift towards person-centred care.

Overall, the intersection of jurisdiction, policy types, and QoL domains confirms that some policies, particularly safety, security and order, may be prioritised in different types of policy document, and in preference to other QoL. Alternatively, the presence of a resident focused QoL orientation in many polices affirms the cultural shift towards greater person-centredness. However, these policies are often less evident and receive less attention in the dominant discourse of policy documents.

The implications of these results are discussed in the next section.

## Discussion

The intersection of the policies used in the approach (Fig. 2), and as described in the findings, points to how policy-makers, LTC decision-makers, and those working in LTC could action policy levers that support QoL for residents. For example, a complementary study [102] explores the enablers of a person-centred culture of change through the meanings of being person-centred in nursing homes. The recognition that policies play out in multiple ways (situational, structural, and temporal), and that policy-driven actions tend to prioritise safety, security, and order, enables the identification of practices that limit the full scope of under-utilised policy directives.

Three policy levers are revealed through the analysis and apply in three key ways: First, resident focused, person-centered QoL policies are currently present in many LTC related policy documents issued by the governments of Canada, British Columbia, Alberta, Ontario, and Nova Scotia, but their expression is not fully realized in the dominant policy discourse. Since this analysis identifies the types of policy and QoL domains that are most vulnerable to restriction by these influences, it maps sites of *potential slippage* for policy makers. These findings are exemplified in an in-depth study of nursing homes [103], indicating that wider socio-economic and political regulations lead to conditions that constrain residents’ independence, individuality, autonomy, preferences, and dignity.

Second, policies relating to quality of care often take precedence over those expressing QoL–this dominance may be expressed explicitly or indirectly in policy documents. As cited earlier, this quality of care/QoL tension has been deconstructed in Canada [22], amongst others. Similarly, with respect to policy language: “By reviewing the legislation, regulations, rules, public forums, and debates; the dominant meanings, assumptions, words, and ideologies can emerge.” [104 p.3].

The importance of paying attention to the nuances embedded in different terminology is also underscored: “Some terms may be degrading or associated with legislation failure. Other terminology may be viewed in a more positive manner commonly associated with approved legislation.” [104 p.5] This analysis extends these perspectives with the notion of *differential policy weights*. Depending on the context and/or prevailing circumstances, different policy elements are assigned preferential significance compared with others. Again, this phenomenon is evident at each of the situational, structural, and temporal stages.

Lastly, the curtailment of many of these *soft* QoL policies is contrary to the *cultural shift* towards person-centered care in Canadian LTC settings. Indicative research in a nursing home with a committed “culture-change” philosophy strengthens this finding by supporting the very necessary resource needs in terms of staff workload required to support QoL for residents [105]. Analogously, although there are instances of person-centeredness in this study, ranging from resident rights in British Columbia to aspirational spiritual wellbeing statements, they are not definitive guarantors of realisation. Despite this uncertainty, the presence of less apparent and/or recently created resident-oriented QoL policies are positive signifiers of this cultural shift.

At this stage, it is necessary to acknowledge that precautionary and preventative policies, such as fire and building codes in LTC facilities, and rules specifying and mandating compliance with safety, security, and order, for example, during the Covid-19 pandemic, are essential. The intent of this analysis is not to downplay these imperatives, but instead, point out that resident-focused QoL expressions already exist, but are dormant in many policies. It also demonstrates and contextualizes examples of policy slippage, differential policy weights, and cultural shifts across existing policies. Accordingly, it offers a timely, positive, and forward-facing roadmap upon which to enhance and build policies that capitalise and enable person-centredness in the provision of LTC in Canada. In essence, it facilitates “reimagining the future in the present.” [106 pp.17–18].

Subsequent to this analysis, it is significant that two of the provinces included in this research have recently legislated new policies for Continuing Care–Ontario [107] and Alberta [108]. They are centered on resident focused care, and based on an informed, comprehensive, and collaborative approach to evidence based, *best* practice. And of particular note, is the 2023 Canadian Long Term Care National Services Standard [109] that focuses on many of the attributes identified in this analysis, such as enabling a meaningful QoL for residents. The Standard’s central tenet equates high quality LTC with care that is resident-centred–signified by two of the six detailed sections, namely, “Upholding Resident-Centred Care” and “Enabling a Meaningful Quality of Life for Residents”. (The remaining sections relate to governance, the workforce, quality of care, and quality improvement.) As such, the Standard provides LTC home teams, leaders, and governing bodies with guidance on providing evidence-informed, resident- care that values compassion, respect, dignity, trust, and a meaningful quality of life, thereby underscoring the soft QoL domains located in this study.

The analytical approach and policy levers identified in this analysis outline a way forward to address both QoL priorities and the management of multiple policies when making decisions about how to act within policy directives. In the post-Covid era where the health system continues to falter under the pressure of providing quality and safe care, seeking existing policies that support person-centred care is a practical and timely activity. Here, the new LTC Standard [109] that embraces this QoL, resident-centered thinking sets the stage for the policy language and resourcing needed to fully realise this imperative. Although the Standard is voluntary for the provincial and territorial jurisdictions (responsible for the delivery of LTC in Canada), it is hoped that it will drive the federal government to transfer monies to jurisdictions and tie this funding to investments and improvements in LTC.

### Limitations

Although the analysis plugs a gap at a macro (policy) level, it does not extend to implementation in practice. Consequently, the findings are directional signifiers rather than definitive *what is* claims. As such, the analysis focuses on exposing hidden and un/under-used policies rather than an examination of the resources required to support their realisation, for instance, funding and staffing.

Similarly, the analysis does not represent all Canadian jurisdictions and LTC related policies–noteworthy omissions include the historical, cultural, and linguistic imperatives of Indigenous and Francophone peoples.

Other limitations relate to the policy library. These include the pre-selection of QoL domains [10, 11] in lieu of alternatives, and the restriction of policies to obligatory *must comply* regulatory power only.

The analysis is also predicated on the expression of policies through language. Without information about the intent of policy makers, terminology is at best, an approximation, and hence, open to different interpretations.

### Questions for further research

These dormant and/or under-utilised policies are presented as potentialities–it is not known if or how they are actually implemented at a practice level.

- Do successive policy supplements strengthen or detract from the original *core* policy?
- Do these additions impact policy robustness and/or contribute to policy fragmentation over time? Are the findings transferable to other jurisdictions and contexts, policy levels and types?
- How does the new Canadian National Services Standard [106] exemplify the QoL domains?

## Conclusion

The analysis provides substantive evidence of person-centredness in many of the current LTC and non-LTC specific policies in Canada. Although these policies are already in existence, they remain dormant and/or under-utilised in the dominant policy discourse. This conclusion is supported with three key policy levers: situations–providing explicit and implicit examples of resident focused QoL policy relegation in each jurisdiction; structures–identifying which types of policy and QoL expressions are more vulnerable to sidelining by others; and trajectories–confirming the cultural shift towards more person-centredness in Canadian LTC related policies over time.

These under-utilised policies are implementation-ready, with the potential to contribute positively to resident QoL in British Columbia, Alberta, Ontario, and Nova Scotia. Again, borrowing from Molinari and Pratt [106 p.3] these “glimmers of hope” were particularly relevant during the Covid-19 pandemic, but they also signal a timely “political possibility” for a post-Covid era, as in the newly created, Canada-wide National LTC Services Standard [109].

## Electronic supplementary material

Below is the link to the electronic supplementary material.

Supplementary Material 1

Supplementary Material 2

Supplementary Material 3

Supplementary Material 4

## Data Availability

The government policies that support the findings of this study are available to the public online and through the links provided in the reference list. The full datasets analyzed in this study are available from the corresponding author on reasonable request.

## Abbreviations

AB: Alberta
BC: British Columbia
LTC: Long-Term Care
NS: Nova Scotia
ON: Ontario
QoL: Quality of Life
QoC: Quality of Care
