# Supplementary material for: Provincial policies affecting resident quality of life in Canadian residential long-term care
Source: BMC Geriatr. 2023 Jun 9;23:362. doi: 10.1186/s12877-023-04074-y (PMC10252178; doi:10.1186/s12877-023-04074-y)
Supplement: Supplementary file 1 — Supplementary Material 1 [file 12877_2023_4074_MOESM1_ESM.docx]

Supplementary Table 1

*Jurisdiction, regulatory level, and relevant quality of life domains*

| **Jurisdiction** | **Policy** | **Kane Domain** |
| --- | --- | --- |
| **Long Term Care Specific Policies** | | |
| **Federal** | No policies |  |
| **British Columbia** | No policies |  |
| **Alberta** | Nursing Homes General Regulation 232 (1985) | Safety, Security, Order (SSO); Privacy |
|  | Nursing Homes Operation Regulation 258 (1985) | SSO; Food/Enjoyment; Meaningful Activity; Relationships; Spiritual Well-Being |
|  | Nursing Homes Act (2000) | SSO; Privacy |
|  | Long-Term Care Accommodation Standards and Checklist (2010) | SSO; Physical Comfort; Food/Enjoyment; Functional Competence; Privacy; Individuality; Spiritual Well-Being |
| **Ontario** | Long-Term Care Homes Act (2007) | SSO; Physical Comfort; Food/Enjoyment; Meaningful Activity; Relationships; Functional Competence; Dignity; Privacy; Individuality; Autonomy; Spiritual Well-Being |
|  | Long-Term Care Home RAI-MDS 2.0 LTC Homes – Practice Requirements (2007) | SSO; Relationships; Functional Competence; Individuality |
|  | Long-Term Care Homes Act Regulation 410/16 (2007) | SSO; Physical Comfort; Food/Enjoyment; Meaningful Activity; Relationships; Functional Competence; Dignity; Privacy; Individuality; Autonomy; Spiritual Well-Being |
|  | Long-Term Care Home Financial Policy Fill Rate Guidelines for New Interim LTC Beds (2010) | SSO |
|  | Long-Term Care Home Financial Policy Registered Practical Nurses in Long-Term Care Homes Initiative Funding Policy (2010) | SSO |
|  | Long-Term Care Homes Funding Policy Funding Policy for Suspension of Admission Due to Outbreaks (2010) | SSO |
|  | Long-Term Care Homes Funding Policy Eligible Expenditures for Long-Term Care Homes (2010) | SSO; Physical Comfort; Relationships; Functional Competence; Individuality |
|  | Long-Term Care Home Policy LTCH Required Goods, Equipment, Supplies and Services (2010) | SSO; Physical Comfort; Food/Enjoyment; Relationships; Functional Competence; Spiritual Well-Being |
|  | Long-Term Care Homes Financial Policy Spousal Supplement for Two-Bed Room Shared by Spouses (2011) | SSO; Relationships; Meaningful Activity; Relationships; Privacy; Individuality; Autonomy; Spiritual Well-Being |
|  | Long-Term Care Home Financial Policy LTCH Cash Flow Policy (2011) | SSO; Individuality |
|  | Long Term Care Homes Funding Policy Behavioural Supports Ontario Staffing Resources (2012) | SSO; Relationships; Functional Competence; Individuality |
|  | Long-Term Care Home Financial Policy LTCH Reconciliation and Recovery Policy (2013) | SSO |
|  | Long-Term Care Homes Financial Policy LTCH Furnishing and Equipment Management (2013) | SSO; Relationships; Individuality |
|  | Long-Term Care Homes Financial Policy LTCH Level-of-Care per Diem Funding Policy (2013) | SSO; Relationships; Functional Competence; Dignity; Individuality |
|  | Long-Term Care Homes Financial Policy LTCH Occupancy Targets Policy (2014) | SSO |
|  | Long-Term Care Homes Funding Policy Attending Nurse Practitioners in Long-Term Care Homes Initiative Funding Policy (2015) | SSO; Meaningful Activity; Relationships; Individuality; Autonomy |
|  | Long-Term Care Home Design Manual (2015) | SSO; Physical Comfort; Food/Enjoyment; Meaningful Activity; Relationships; Functional Competence; Dignity; Privacy; Individuality; Autonomy; Spiritual Well-Being |
|  | Construction Funding Subsidy Policy for Long-Term Care Homes (2015) | Physical Comfort; Food/Enjoyment; Meaningful Activity; Relationships; Functional Competence |
|  | Long-Term Care Home Financial Policy LTCH Physiotherapy Funding Policy (2016) | SSO; Relationships; Functional Competence; Individuality |
|  | Long-Term Care Homes Financial Policy LTCH Convalescent Care Additional Subsidy Funding Summary (2016) | Functional Competence; Individuality |
|  | Long-Term Care Homes Funding Policy LTCH Bad Debt Reimbursement (2016) | SSO; Relationships |
| **Nova Scotia** | Homes for Special Care Act (1989) | SSO; Food/Enjoyment; Meaningful Activity; Relationships; Dignity; Autonomy |
|  | Long Term Care Facility Requirements (Space and Design) (2007) | SSO; Physical Comfort; Food/Enjoyment; Meaningful Activity; Relationships; Functional Competence; Dignity; Privacy; Individuality; Autonomy; Spiritual Well-Being |
|  | Special Needs Policy – Long Term Care (2008) | SSO |
|  | Unprotected Envelope Funding Policy (2008) | SSO; Relationships; Individuality |
|  | Over Cost Fund Policy (2008) | SSO; Relationships; Functional Competence; Individuality; Autonomy |
|  | Resident Trust Account Policy (2009) | SSO; Relationships; Functional Competence; Autonomy |
|  | Homes for Special Care Regulations (2012) | SSO; Physical Comfort; Food/Enjoyment; Meaningful Activity; Relationships; Functional Competence; Dignity; Individuality; Autonomy; Spiritual Well-Being |
|  | Nursing Home Maintenance Standard (2013) | SSO; Physical Comfort |
|  | HELP-Specialized Equipment Program Guidelines (2014) | SSO; Relationships; Functional Competence |
|  | Long Term Care Program Requirements: Nursing Homes & Residential Care Facilities (2016) | SSO; Physical Comfort; Food/Enjoyment; Meaningful Activity; Relationships; Functional Competence; Dignity; Individuality; Autonomy; Spiritual Well-Being |
|  | Resident Charge Policy (2016) | SSO; Physical Comfort; Food/Enjoyment; Meaningful Activity; Relationships; Functional Competence; Privacy; Individuality; Autonomy |
| **Non-Long-Term Care Specific Policies** | | |
| **Federal** | The Constitution Acts 1867 to 1982 (2013) | SSO |
|  | Canada Health Act (1985) | SSO |
|  | Employment Insurance Act (1996) | SSO |
|  | Personal Information Protection and Electronic Documents Act (2000) | SSO |
|  | Veterans Affairs Canada Residential Care Policy (2012) | SSO; Relationships |
|  | Veterans Affairs Canada Palliative Care Policy (2012) | SSO; Relationships; Individuality; Autonomy; Spiritual Well-Being |
|  | Employment Insurance Act Compassionate Care Benefit (2016) | SSO; Relationships |
|  | Statutes of Canada Chapter 3 An Act to Amend the Criminal Code and to Make Related Amendments to Other Acts (Medical Assistance in Dying) (2016) | Autonomy |
| **British Columbia** | Representation Agreement Act [RSBC 1996] Chapter 405 (1996) | SSO; Physical Comfort; Functional Competence; Dignity; Privacy; Individuality; Autonomy; Spiritual Well-Being |
|  | Mental Health Act [RSBC 1996] Chapter 288 (1996) | SSO |
|  | Workers Compensation Act [RSBC 1996] Chapter 492 (1996) | SSO |
|  | Workers Compensation Act Occupational Health and Safety Regulation 296/97 (1996) | SSO |
|  | Workers Compensation Act Reports of Injuries Regulation 713/74 (1974) | SSO |
|  | Model Standards for Continuing Care and Extended Care Services (1999) | SSO; Physical Comfort; Food/Enjoyment; Meaningful Activity; Relationships; Individuality; Autonomy; Spiritual Well-Being |
|  | Pharmacy Operations and Drug Scheduling Act [SBC 2003] Chapter 77 (2003) | SSO |
|  | Community Care and Assisted Living Act Residential Care Regulation 96 (2009) | SSO; Physical Comfort; Food/Enjoyment; Meaningful Activity; Relationships; Dignity; Privacy; Individuality; Autonomy; Spiritual Well-Being |
|  | Residents' Bill of Rights (n.d.) | SSO |
|  | Seniors Advocate Act [SBC] Chapter 15 (2013) | SSO |
|  | Home and Community Care Policy Manual Chapter 6 Residential Care Services (2016) | SSO; Physical Comfort; Food/Enjoyment; Meaningful Activity; Functional Competence; Dignity; Autonomy |
| **Alberta** | Duties and Reporting Under the Protection for Persons in Care Act (2012) | SSO |
|  | Design Guidelines for Continuing Care Facilities in Alberta (2014) | SSO; Physical Comfort; Food/Enjoyment; Meaningful Activity; Dignity; Privacy; Individuality |
|  | Accommodation Standards and Licensing Information Guide (2015) | SSO; Physical Comfort; Food/Enjoyment; Meaningful Activity; Dignity; Privacy; Individuality; Autonomy |
|  | Continuing Care Health Service Standards (2016) | SSO; Individuality; Autonomy |
|  | Occupational Health and Safety Act (2017) | SSO |
|  | Resident and Family Councils Act (2017) | SSO; Meaningful Activity; Relationships; Privacy; Individuality; Autonomy |
| **Ontario** | Occupational Health and Safety Act  Regulation 67/93 Health Care and Residential Facilities (1990) | SSO |
|  | Occupational Health and Safety Act (1990) | SSO |
|  | Workplace Safety and Insurance Act (1997) | SSO |
|  | Fire Protection and Prevention Act (1997) | SSO |
|  | Fire Protection and Prevention Act Regulation 213/07 (1997) | SSO; Relationships |
|  | Fire Protection and Prevention Act Regulation 364/13 (1997) | SSO; Relationships |
|  | Employment Standards Act (2000) | Relationships |
|  | Patient Restraints Minimization Act (2001) | SSO; Relationships; Dignity; Autonomy; Spiritual Well-Being |
|  |  |  |
|  | Accessibility for Ontarians with Disabilities Act (2005) | SSO |
|  | Accessibility for Ontarians with Disabilities Act Regulation 191/11 (2005) | SSO; Physical Comfort; Food/Enjoyment; Relationships; Functional Competence; Dignity; Individuality |
|  | Excellent Care for All Act (2010) | SSO; Meaningful Activity; Relationships; Privacy; Autonomy |
|  | Excellent Care for All Act Regulation 188/15 Patient Relations Process (2010) | SSO; Meaningful Activity; Relationships |
|  | Excellent Care for All Act Regulation 187/15 Annual Quality Improvement Plan (2010) | Meaningful Activity; Relationships; Autonomy |
|  | Building Code Act Regulation 332/12 (1992) | SSO; Functional Competence |
| **Nova Scotia** | Activities Designation Regulations made under Section 66 of the Environment Act (2016) | SSO; Food/Enjoyment; Autonomy |
|  | Occupational Health and Safety Act (1996) | SSO; Relationships |
|  | Smoke Free Places Act (2002) | SSO; Individuality; Autonomy |
|  | Fire Safety Act (2002) | SSO; Relationships |
|  | Safer Needles in Healthcare Workplaces Act (2006) | SSO |
|  | Occupational Health and Safety Act Violence in the Workplace Regulations 209/2007 (2007) | SSO; Relationships |
|  | Personal Directives Act (2008) | SSO; Functional Competence; Autonomy; Spiritual Well-Being |
|  | Building Code Regulations (2017) | SSO; Functional Competence; Autonomy |
|  | Fire Safety Regulation 68/2017 (2017) | SSO |
