# Supplementary material for: Provincial policies affecting resident quality of life in Canadian residential long-term care
Source: BMC Geriatr. 2023 Jun 9;23:362. doi: 10.1186/s12877-023-04074-y (PMC10252178; doi:10.1186/s12877-023-04074-y)
Supplement: Supplementary file 2 — Supplementary Material 2 [file 12877_2023_4074_MOESM2_ESM.docx]

Supplementary Table 2

*Long-term care specific policies ordered by regulatory type, jurisdiction, and date*

| **Jurisdiction** | **Regulatory Type and Policy Name** |
| --- | --- |
|  | **Legislation/Regulation** |
| **Alberta** | Nursing Homes Act (2000) including: General Regulation 232; Operation Regulation 258 |
| **Ontario** | Long Term Care Homes Act (2007) including: RAI MDS 2.0 LTC Homes – Practice Requirements; Regulation 410/16  **Long-Term Care Homes LTCH Financial Policy (2010)** including:  Fill Rate Guidelines for New Interim Beds; Suspension of Admission Due to Outbreaks; Registered Practical Nurses in Long-Term Care Initiative; Eligible Expenditures for Long-Term Care Homes; Furnishing and Equipment Management; Required Goods, Equipment, Supplies and Services  **Long-Term Care Homes LTCH Financial Policy (2011)** including:  Cash Flow; Spousal Supplement for Two-Bed Room Shared by Spouses  **Long-Term Care Homes LTCH Financial Policy (2012)** including:  Behavioural Supports Ontario Staffing Resources  **Long-Term Care Homes LTCH Financial Policy (2013)** including:  Reconciliation and Recovery Policy; Level of Care-per-Diem Funding Policy  **Long-Term Care Homes LTCH Financial Policy (2014)** including:  Occupancy Targets Policy  **Long-Term Care Homes LTCH Financial Policy (2015)** including:  Attending Nurse Practitioners in Long-Term Care Homes Initiative Funding Policy  **Long-Term Care Homes LTCH Financial Policy (2016)** including:  Physiotherapy Funding Policy; Bad Debt Reimbursement; Convalescent Care Additional Subsidy Funding Summary |
| **Nova Scotia** | Unprotected Envelope Funding Policy (2008)  Homes for Special Care Act (1989) including: Homes for Special Care Regulations (2012)  Long Term Care Facility Requirements (Space and Design) (2007)  Long-Term Care Program Requirements: Nursing Homes & Residential Care Facilities (2016) |
|  | **Standards** |
| Alberta | Long-Term Care Accommodation Standards and Checklist (2010) |
| Nova Scotia | Nursing Home Maintenance Standard (2013) |
|  | **Manuals** |
| Ontario | Long-Term Care Home Design Manual (2015) |
| Nova Scotia | Special Needs Policy – Long Term Care (2008)  Over Cost Fund Policy (2008)  Resident Trust Account Policy (2009)  HELP-Specialized Equipment Program Guidelines (2014)  Resident Charge Policy (2016) |

**Notes**

1. Legislation/Regulation: No examples in British Columbia or Federally

2. Standards: No examples in British Columbia, Ontario, or Federally

3. Manuals: No examples in Alberta, British Columbia, or Federally.
