# Supplementary material for: Provincial policies affecting resident quality of life in Canadian residential long-term care
Source: BMC Geriatr. 2023 Jun 9;23:362. doi: 10.1186/s12877-023-04074-y (PMC10252178; doi:10.1186/s12877-023-04074-y)
Supplement: Supplementary file 3 — Supplementary Material 3 [file 12877_2023_4074_MOESM3_ESM.docx]

Supplementary Table 3

*Overarching non-long-term care specific policies ordered by regulatory type, jurisdiction, and date*

| **Jurisdiction** | **Regulatory Type and Policy Name** |
| --- | --- |
|  | **Legislation/Regulation** |
| **Federal** | The Constitutions Act 1867 to 1852 (2013)  Canada Health Act (1985)    Employment Insurance Act (1996) including: Compassionate Care Benefit (2016)  Personal Information Protection and Electronic Documents Act (2000)  Veterans Affairs Canada (2012) including: Residential Care Policy; Palliative Care Policy    Statutes of Canada Chapter 3 An Act to Amend the Criminal Code and to Make Related Amendments to Other Acts (Medical Assistance in Dying) (2016) |
| **British Columbia** | Representation Agreement Act [RSBC 1996] 405 (1996)  Pharmacy Operations and Drug Scheduling Act [SBC 2003] 77 (2003)  Seniors Advocate Act [SBC] 15 (2013)  Workers Compensation Act [RSBC 1996] 492 (1996) including: Reports of Injuries Regulation 713/74; Occupational Health and Safety Regulation 296/97  Community Care and Assisted Living Act Residential Care Regulation 96 (2009)  Residents' Bill of Rights (n.d.) |
| **Alberta** | Duties and Reporting Under the Protection for Persons in Care Act (2012)  Occupational Health and Safety Act (2017)  Resident and Family Councils Act (2017) |
| **Ontario** | Occupational Health and Safety Act (1990) including: Regulation 67/93 Health Care and Residential Facilities  Building Code Act Regulation 332/12 (1992)  Workplace Safety and Insurance Act (1997)  Fire Protection and Prevention Act (1997) including: Fire Code Regulation 213/07; 364/13  Employment Standards Act (2000)  Patient Restraints Minimization Act (2001)  Accessibility for Ontarians with Disabilities Act (2005) including: Regulation 191/11  Excellent Care for All Act (2010) including: Regulation 187/15; Regulation 188/15 |
| **Nova Scotia** | Occupational Health and Safety Act (1996) including: Violence in the Workplace Regulation 209/2007 (2007)  Smoke Free Places Act (2002)  Fire Safety Act (2002) including: Regulation 68/2017 (2017)  Safer Needles in Healthcare Workplaces Act (2006)  Personal Directives Act (2008)  Building Code Regulations (2017)  Activities Designation Regulations made under Section 66 of the Environment Act (2016) |
|  | **Standards^1^** |
| **Alberta** | Continuing Care Health Service Standards (2016) |
|  | **Manuals^2^** |
| **British Columbia** | Home and Community Care Policy Manual Chapter 6 Residential Care Services (2016) |
|  | **Guidelines^3^** |
| **Alberta** | Design Guidelines for Continuing Care Facilities in Alberta (2014)  Accommodation Standards and Licensing Information Guide (2015) |

**Notes**

^1^ Standards: No examples in British Columbia, Ontario, Nova Scotia, or Federally

^2^ Manuals: No examples in Alberta, Ontario, Nova Scotia, or Federally

^3^ Guidelines: No examples in British Columbia, Ontario, Nova Scotia, or Federally
