# Supplementary figures and images for: Provincial policies affecting resident quality of life in Canadian residential long-term care
Source: BMC Geriatr. 2023 Jun 9;23:362. doi: 10.1186/s12877-023-04074-y (PMC10252178; doi:10.1186/s12877-023-04074-y)

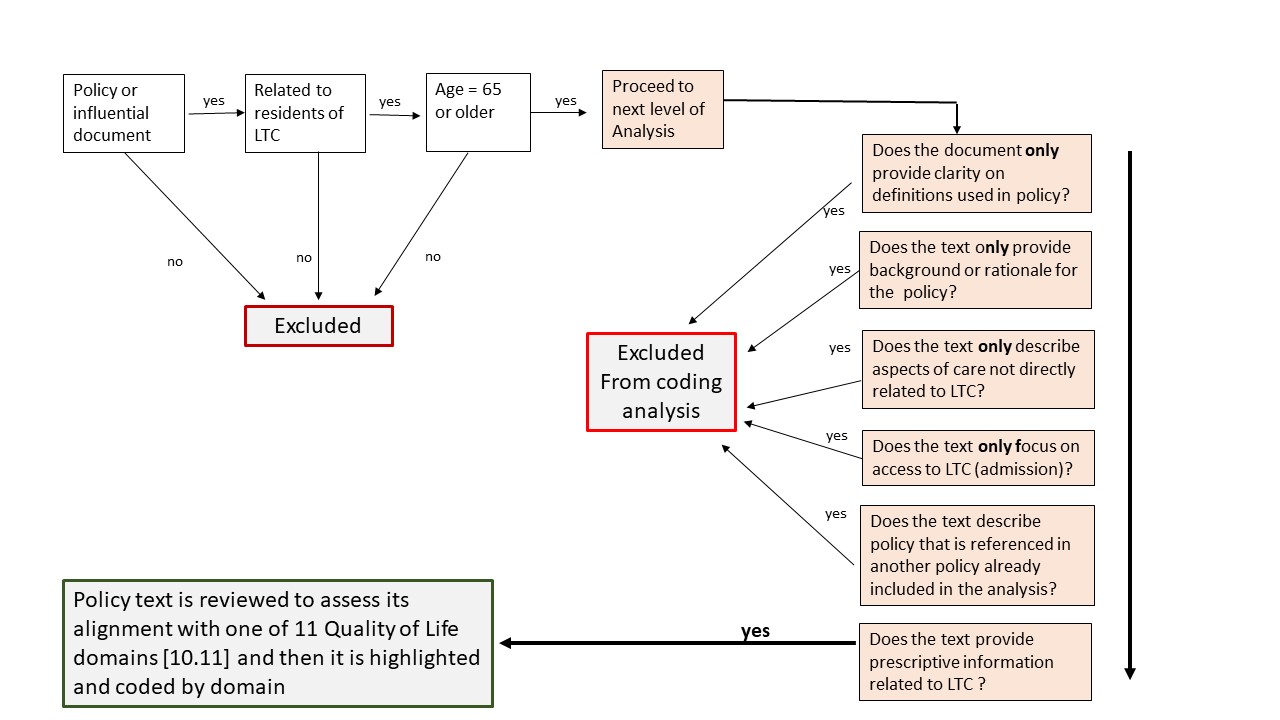

Supplement: Supplementary file 4 — Supplementary Material 4 [file 12877_2023_4074_MOESM4_ESM.jpg]
